# New Trends and Technologies in Modern Neurosurgery

**DOI:** 10.3390/brainsci16070714

**Published:** 2026-07-03

**Authors:** Massimiliano Visocchi, Francesco Signorelli

**Affiliations:** 1 Department of Neurosurgery, Fondazione Policlinico Universitario A. Gemelli IRCCS, Italy Catholic University, Italy Largo Agostino Gemelli 8, 00168 Rome, Italy; massimiliano.visocchi@unicatt.it; 2 Department of Neurosurgery, Università Cattolica del Sacro Cuore, 00168 Rome, Italy

The neuraxis—the skull base along with its offshoot, the spine—replicates a bone funnel as a vessel, sustaining the brain, the cerebellum and the spinal cord, along with cranial and radicular nerves. Knowledge of the embryology, anatomy, physiology and pathophysiology of diseases and of more effective surgical pathways for engaging with and eliminating surgical diseases is of paramount importance in surgical cultural heritage and should be strongly encouraged and supported in young neurosurgeons. New trends and technologies are growing quickly and effectively; thus, the aim of this Special Issue is to raise awareness about their use in modern neurosurgical practice.

The spirit we promoted in this Special Issue of *Brain Sciences* was driving the practical interests of neurosurgeons in further investigating and implementing such emerging new trends in technologies, both in research and in surgical practice.

New trends manuscripts, never published previously, were submitted online; all of them that passed pre-checking were peer-reviewed. The papers were published in the journal as soon as they were accepted and are listed together on the Special Issue website. Research articles, review articles, and short communications were invited to apply. After the submission of neurosurgically tailored papers, we realized that the papers accepted for publication were able to make clear reference to new trends and technologies in this challenging field.

We published 13 papers in the first edition and five papers in the second. The main topics and questions that arose in the issue are outlined below.

## 1. Surgical Tools and Intraoperative Facilities

Augmented reality (AR) and mixed reality (MR), along with artificial intelligence (AI) technologies, have revolutionized cranial and spine neurosurgery by overlaying digital information onto the surgical field, enhancing visualization, precision and training. Studies have demonstrated the successful use of AR, MR and AI in surgery, enhancing precision, clinical outcomes and education, despite hurdles such as technical limitations and integration challenges. In particular, AR’s immersive visualizations and educational innovations, coupled with its potential synergy with AI and machine learning, seem to indicate a bright future for surgical care. The exoscope has also made great progress in innovation, with continuous demand in the field of visual enhancement technologies and video streaming. The discovery of new systems capable of improving the visualization and illumination of the surgical field, such as the exoscope, has allowed these technologies to become part of the neurosurgical routine [1,2,3].

## 2. Spine

The neurosurgical treatment of thoracic disc herniation (TDH) has undergone dramatic changes over the years in terms of surgical approaches and intraoperative technological tools. There is still no unanimous consent on the criteria for approach selection, and the choice varies among institutions. Posterolateral approaches offer advantages in terms of safety, recovery, neurological improvement, and complete hernia resection. Surgical spine decompression seems to be better characterized by the change in neurophysiological properties at the cortical and corticospinal level. The surgical decompression of the myelopathic spinal cord improves the neurophysiological balance at the cortical and corticospinal level, resulting in clinically significant recovery. Such findings contribute to the existing evidence characterizing the brain and the spinal cord as a dynamic system capable of functional and reversible plasticity, and provide useful clinical insights to be used for patient counseling. Moreover, as is well known, lumbar foraminal stenosis (LFS) involves the narrowing of neural foramina, leading to nerve compression, significant lower back pain and radiculopathy, particularly in the aging population. The comprehensive review published in this Special Issue underscores the necessity for precise diagnostic and management strategies for LFS, highlighting the role of a multidisciplinary approach and the utility of a unified classification system in enhancing patient outcomes in the face of this condition’s increasing prevalence. Finally, advancements in spine neurosurgical training, especially with the use of low-cost 3D models for simulating cervical spine tumor removal, are revolutionizing this field. These models provide the realistic and hands-on experience crucial for mastering complex neurosurgical techniques, filling gaps left by traditional educational methods. Nevertheless, continuing laboratory training for residents remains crucial [4,5,6,7].

## 3. Tumors

Glioblastoma (GBM) is an extremely aggressive brain tumor that has few available treatment options and a dismal prognosis. Recent research has highlighted the potential of extracellular vesicles (MSC-EVs) produced from mesenchymal stem cells as a potential treatment approach for GBM. Nanomedicine, which involves nanoparticles in the 1–100 nm range, shows promise in improving drug delivery and the targeting of tumor cells. It offers a modest increase in survival with fewer severe side effects. It shows promise in improving quality of life, especially in recurrent GBM patients. However, it falls short in terms of overall survival compared to Bevacizumab. The heterogeneous nature of treatment protocols and reporting methods highlights the need for standardized multicenter trials to further evaluate the potential of nanomedicine in GBM management. On the other hand, gangliogliomas are rare tumors accounting for about 0.4% of all central nervous system tumors. They are usually located in the temporal lobes of children and young adults, though such tumors in the infratentorial region and in adult-age patients are rarely reported. Infratentorial glioneuronal tumors are rare findings in the adult population, and histopathological characterization does not seem to fully reflect their true behavior. Future studies are warranted for better characterizing histopathological findings and treatment [8,9,10].

## 4. CSF Dynamics

Spontaneous intracranial hypotension (SIH) in Marfan syndrome and its management are also discussed in this Special Issue. Patients with connective tissue disorders such as MS are at an increased risk for SIH. Few studies have assessed the management of these patients, but different strategies investigated here and in the available literature have provided further interesting data [11].

## 5. Trauma

Females are at greater risk and experience SRC differently to males; this is most likely due to a combination of biomechanical factors, differences in neck musculature, and hormonal and social factors. Sex differences are not widely addressed by the 6th ICSS, which informs many sport-associated concussion protocols [12].

## 6. Chiari

Chiari malformation type 1 (CM1) remains a complex neurosurgical condition with ongoing debate regarding its optimal management. The need for individualized treatment plans for CM1 is emphasized, with special focus placed on the connection between novel pathophysiological insights, technological advancements and opportunities for a more nuanced surgical management. Chiari formation or malformation? This is the issue [13].

In conclusion, this interesting heterogeneous collection of papers on the modern neurosurgery landscape seems to fulfill the target of this Special Issue on new trends and technologies in modern neurosurgery, with a view to expanding their use in the near future.
